# Interleukin‐2 induces extracellular matrix synthesis and TGF‐β2 expression in retinal pigment epithelial cells

**DOI:** 10.1111/dgd.12630

**Published:** 2019-10-13

**Authors:** Ruihua Jing, Tiantian Qi, Chan Wen, Jiaqi Yue, Guangyan Wang, Cheng Pei, Bo Ma

**Affiliations:** ^1^ Department of Ophthalmology The First Affiliated Hospital of Xi'an Jiaotong University Xi'an China

**Keywords:** age‐related macular degeneration, epithelial‐mesenchymal transition, extracellular matrix synthesis, Interleukin 2, retinal pigment epithelial cells

## Abstract

Macular fibrosis is a vital obstacle of vision acuity improvement of age‐related macular degeneration patients. This study was to investigate the effects of interleukin 2 (IL‐2) on epithelial‐mesenchymal transition (EMT), extracellular matrix (ECM) synthesis and transforming growth factor β2 (TGF‐β2) expression in retinal pigment epithelial (RPE) cells. 10 μg/L IL‐2 was used to induce fibrosis in RPE cells for various times. Western blot was used to detect the EMT marker α‐smooth muscle actin (α‐SMA), ECM markers fibronectin (Fn) and type 1 collagen (COL‐1), TGF‐β2, and the activation of the JAK/STAT3 and NF‐κB signaling pathway. Furthermore, JAK/STAT3 and NF‐κB signaling pathways were specifically blocked by WP1066 or BAY11‐7082, respectively, and the expression of α‐SMA, COL‐1, Fn and TGF‐β2 protein were detected. Wound healing and Transwell assays were used to measure cell migration ability of IL‐2 with or without WP1066 or BAY11‐7082. After induction of IL‐2, the expressions of Fn, COL‐1, TGF‐β2 protein were significantly increased, and this effect was correlated with IL‐2 treatment duration, while α‐SMA protein expression did not change significantly. Both WP1066 and BAY11‐7082 could effectively downregulate the expression of Fn, COL‐1 and TGF‐β2 induced by IL‐2. What's more, both NF‐κB and JAK/STAT3 inhibitors could suppress the activation of the other signaling pathway. Additionally, JAK/STAT3 inhibitor WP1066 and NF‐κB inhibitor BAY 11‐7082 could obviously decrease RPE cells migration capability induced by IL‐2. IL‐2 promotes cell migration, ECM synthesis and TGF‐β2 expression in RPE cells via JAK/STAT3 and NF‐κB signaling pathways, which may play an important role in proliferative vitreoretinopathy.

## Graphical abstract

The study presented the non‐canonical effects of IL‐2 on the migration, mechanisms of extracellular matrix synthesis as well as the effects of TGF‐β2 expression in RPE cells via NF‐κB and JAK/STAT3 signaling pathway.

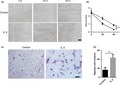

## 1 INTRODUCTION

As a major cause of severe loss of vision among the old, age‐related macular degeneration (AMD) is a complex multifactorial disease clinically caused by the degeneration of the photoreceptors and retinal pigment epithelium (RPE) cells in the old (Yonekawa & Kim, 2014). AMD is broadly classified into the wet form (neovascular or exudative) and dry form. Both two types of AMD pathology start with the formation of insoluble aggregates and drusen, which forms in the extracellular matrix between Brush's membrane and RPE (Mitchell, Liew, Gopinath, & Wong, 2018). Nowadays the main therapeutic medication of neovascular age‐related macular degeneration (nAMD)‐ anti‐vascular endothelial growth factor (VEGF)—includes bevacizumab and ranibizumab (Solomon, Lindsley, Vedula, Krzystolik, & Hawkins, 2019). Delightedly, the treatment could prevent the decrease or even improve vision acuity. However, after frequently, costly and invasive intravitreal injections, up to one‐third of patients could not benefit from the therapy due to the development of macular fibrosis or atrophy (Little, Ma, Yang, Chen, & Xu, 2018).

Considering TGF‐β is a dominant factor in the development of AMD and macular fibrosis or atrophy (Wang et al., 2019), the mechanism of enhanced TGF‐β activity after anti‐VEGF by intravitreal injection may provide a new way to prevent the development of nAMD and macular fibrosis or atrophy. While inflammation is an inevitable response after intravitreal injection and it plays important roles in nAMD (Kauppinen, Paterno, Blasiak, Salminen, & Kaarniranta, 2016; Rezar‐Dreindl et al., 2016; Roh et al., 2009). IL‐2, a characteristic inflammatory factor, plays major roles in many eye diseases and is involved in fibrosis in many tissues (Jung, Woo, & Park, 2019; Wang, Wang, Zhu, Geng, & Yang, 2015). The expression level of IL‐2 has been found to be highly expressed in the aqueous humour of polypoidal choroidal vasculopathy (PCV) and proliferative vitreoretinopathy (PVR) patients (Ricker et al., 2011; Roybal et al., 2018; Sakurada et al., 2015). In addition, the inflammation pathways associated to IL‐2 were activated in AMD (Makarev et al., 2014; Newman et al., 2012). Various studies have confirmed that IL‐2 could interact with TGF‐β to enhance their activities (Battaglia et al., 2013; Chambers et al., 2014; Freudenberg et al., 2018; Tischner, Wiegers, Fiegl, Drach, & Villunger, 2012). It is speculated that persistent activation of IL‐2 may be an important contributor to the increase of TGF‐β effects. However, whether IL‐2 could directly induce the occurrence of EMT and synthesis of ECM and whether there was a connection between TGF‐β activity and IL‐2 in RPE cells remain unknown.

Numerous studies have elucidated various functions of JAK/STAT3 signaling pathway related to ROS, inflammatory, immune and so on in AMD pathogenesis (Fasler‐Kan, Wunderlich, Hildebrand, Flammer, & Meyer, 2005; Kutty et al., 2018; Lin et al., 2013; Yamamoto, Fara, Dasgupta, & Kemper, 2013). While JAK/STAT3 signaling pathway is validated to cause EMT or ECM synthesis in multiple tissue or diseases (Kim et al., 2019; Li et al., 2018). Meanwhile, NF‐κB signaling pathway was also proved to work in AMD or EMT or ECM synthesis in other disease (Hseu et al., 2019; Shen, Xie, Gu, Li, & Tong, 2016; Zhang & Huang, 2018). Additionally, cytokines like interleukin 6 (IL‐6) were found to activate JAK/STAT3 and NF‐κB signaling pathways (Hendrayani, Al‐Harbi, Al‐Ansari, Silva, & Aboussekhra, 2016; Li, Ye, Huang, Zhang, & He, 2019). Whereas the relationship of IL‐2 and JAK/STAT3 and NF‐κB pathways is not clear in nAMD.

According to these effects of IL‐2 and fibrosis in AMD, we hypothesized that IL‐2 may serve as an inducer function by interacting with TGF‐β and activating certain pathways. To validate, we simulated the inflammatory microenvironment of the AMD with IL‐2 in this study, then used Western blot method to detect the EMT marker, the ECM markers, and the activation of the JAK/STAT3 and NF‐κB signaling pathway. In addition, Wound healing and Transwell assays were enrolled to detect cell migration capability. We found that IL‐2 could promote RPE cells migration, ECM markers fibronectin (Fn) and type 1 collagen (COL‐1), TGF‐β2 but not EMT marker α‐smooth muscle actin (α‐SMA) depending on the activation of JAK/STAT3 and NF‐κB signaling pathways.

## 2 MATERIALS AND METHODS

### 2.1 Culture and treatment of retinal pigment epithelial cells

The ARPE19 cell line was purchased from ATCC. A total of 1 × 10^6^ cells within 12 passages were seeded into a culture flask with DMEM/F12 containing 10% fetal bovine serum (FBS) and 1% penicillin‑streptomycin. The medium was renewed by serum‐free DMEM/F12 when the cells get to 70% confluence, and then cells were treated with IL‐2 for different durations (0, 12, 24, 36 and 48 hr) or at different concentrations (0, 0.5, 1, 2, 4, 6 and 8 μg/L). Cells in the control group were treated with an equal volume of medium.

### 2.2 Western blot

Western blot was performed as described in our previous study (Ma, Yang, et al., 2018). Briefly, RPE cell lysis was loaded at a concentration of 20 μg per well to separate in SDS‐PAGE and transferred to polyvinylidene difluoride membranes (Bio‐Rad). The membranes were blocked using 1% BSA (Beijing Biodee Biotechnology Co., Ltd) in tris‐buffered saline containing 0.1% Tween‐20 for 1 hr at room temperature and probed using antibodies shown in Table 1 overnight at 4°C. After incubation with appropriate secondary antibodies, protein signals were detected using enhanced chemiluminescence western blotting detection kit (Bio‐Rad).

**Table 1:** List of various antibodies used for Western blot

Antibody	Dilution	Source
Mouse anti‐α‐SMA	1:1,000	ab5694, Abcam, Cambridge, MA
Rabbit anti‐Fn	1:1,000	ab2413, Abcam, Cambridge, MA
Rabbit anti‐COL‐1	1:200	AB745, Millipore, Billerica, MA
Rabbit anti‐TGF‐β2	1:1,000	ab36495, Abcam, Cambridge, MA
Rabbit anti‐p‐STAT3	1:1,000	#9145, Cell Signaling, Danvers, MA
Rabbit anti‐STAT3	1:1,000	#12640, Cell Signaling, Danvers, MA
Rabbit anti‐IκBα	1:1,000	12045, Sino Biological, Beijing, China
Rabbit anti‐p‐IκBα	1:1,000	#9246, Cell Signaling, Danvers, MA
Rabbit anti‐β‐actin	1:1,000	#4970, Cell Signaling, Danvers, MA
Goat anti‐rabbit IgG secondary antibody	1:3,000	401315, Millipore, Billerica, USA
Goat anti‐mouse IgG secondary antibody	1:3,000	401215, Millipore, Billerica, USA

### 2.3 Wound healing and Transwell^®^ migration assay

Wound healing and Transwell^®^ migration assay were performed as described in our previous study (Ma, Jing, et al., 2018). The cells were in serum‐free medium containing 10 μg/L IL‐2 with or without WP1066 (S2796; Selleck) or BAY 11‐7082 (S2913; Selleck).

### 2.4 Cell viability detection using CCK8 kit

Approximately 10,000 cells were seeded in a 96 well plate. The medium was replaced with serum‐free medium containing IL‐2 and renewed 24 hr later. Cells were treated with different time (0, 24 and 48 hr) and concentration (0, 1, 5, 10, 15 and 20 μg/L) grades. After induction, 100 μl/ml CCK8 solution in medium was added into the microplate. 4 hr later, absorbance at 450 nm and relevance at 650 were measured using a microplate reader.

### 2.5 Statistical analysis

GraphPad Prism 6.0 (GraphPad Software, Inc.) was employed to perform all of the statistical analyses. All of the data are reported as the mean ± *SEM* with at least three independent experiments. Statistical comparisons of the Western blot data were analyzed using one‐way ANOVA, whereas differences between groups were compared using Tukey's honest significant difference (HSD) test. Differences of *p *<* *.05 were considered statistically significant.

## 3 RESULTS

### 3.1 IL‐2 stimulates RPE cells migrate

**Figure 1:**
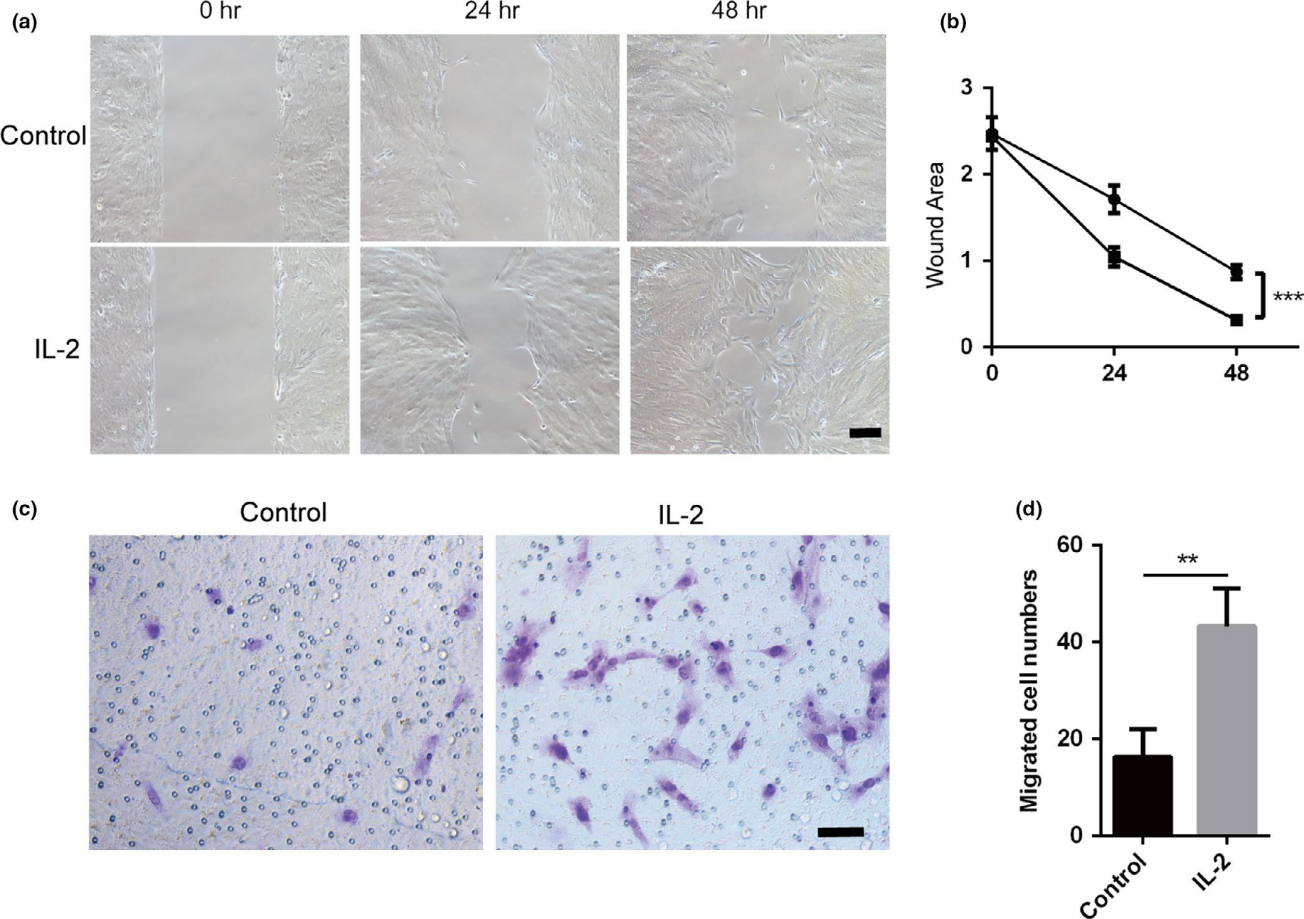
IL‐2 promoted RPE cells migration. (a) RPE cells were treated by 10 μg/L IL‐2 when cells were in 70% confluence after the insert was removed in serum‐free medium. Wound healing at 0, 24 and 48 hr. (b) The wound area was measured and analyzed with ImageJ software and there was a significance in 48 hr between the control group and IL‐2 treated group (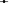, Control; 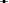, IL‐2). (c) Transwell assay between control group and 10 μg/L IL‐2 treated group after cells were seeded in the Transwell chamber 24 hr later. (d) Migrated RPE cells in control group and IL‐2 group, respectively. **p <* *.01, ***p <* *.001. Scar bar: 100 μm

Varies cytokines were verified to be involved in fibrosis disease among which IL‐2 was also discovered to function in prolapsed lumbar intervertebral disc and hypertension after glaucoma surgery (Jung et al., 2019; Wang et al., 2015). Whether IL‐2 had the analogous effect was unknown on RPE cells. We first investigated the impact of IL‐2 on the migration ability of RPE cells using Transwell and wound healing assays. The results showed that the relative wound area was much less in the IL‐2 treated group than the control group (*p *<* *.05, Figure 1a,b). Similar results were also observed in the Transwell assay (*p *<* *.01, Figure 1c,d). The results indicated that IL‐2 promoted the migration capability of RPE cells in vitro.

### 3.2 IL‐2 promotes ECM synthesis and TGF‐β2 expression in RPE cells

**Figure 2:**
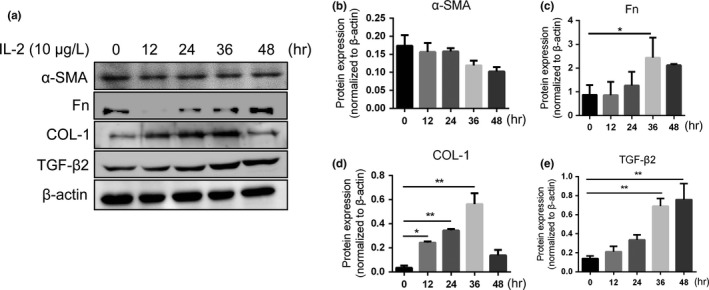
IL‐2 promoted RPE cells ECM synthesis and TGF‐β2 expression. (a) RPE cells were treated by 10 μg/L IL‐2 for 0, 12, 24, 36 and 48 hr, α‐SMA, COL‐1, Fn and TGF‐β2 protein expression were detected by Western blot. (b–e) Quantification of the Western blot analysis results. IL‐2 treatment increased COL‐1, Fn and TGF‐β2 protein expression but not α‐SMA time dependently. *p < .05, **p < .01

Previous studies have shown that IL‐2 could promote cell migration and ECM synthesis in nucleus pulposus cells (Wang et al., 2015). However, the ability of IL‐2 to promote EMT and ECM synthesis in RPE cells was not clear. Our results showed that after treating RPE cells with IL‐2 for various lengths of time (0, 12, 24, 36 and 48 hr), the protein expression levels of COL‐1, Fn and TGF‐β2 were significantly upregulated in a time‐dependent manner (Figure 2a,c–e). However, the expression of α‐SMA was unchanged after treatment with different concentrations of IL‐2 for various durations of time (Figure 2b). Therefore, the results indicate that IL‐2 could promote ECM synthesis but not EMT in RPE cells.

### 3.3 Role of the JAK/STAT3 and NF‐κB signaling pathway in ECM synthesis in RPE cells treated with IL‐2

**Figure 3:**
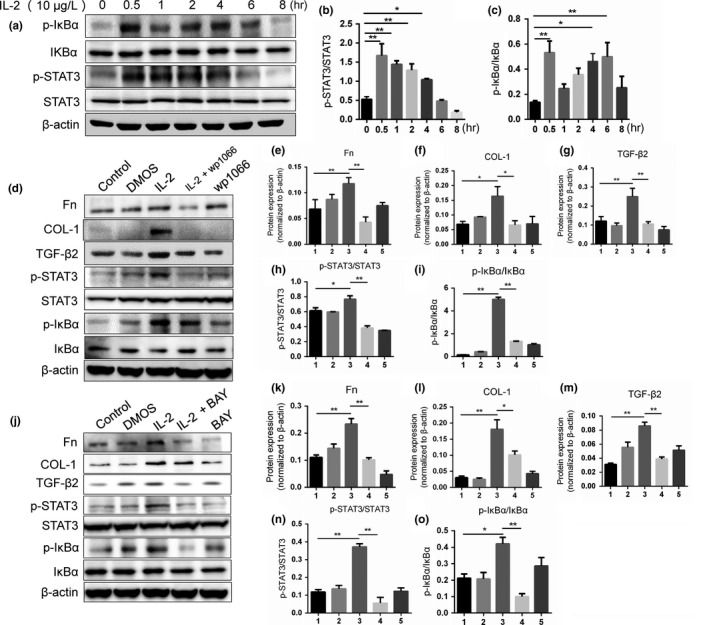
Both JAK/STAT3 and NF‐κB signalling pathway involved in ECM synthesis and TGF‐β protein expression. (a) RPE cells were treated by 10 μg/L IL‐2 for different duration (0, 0.5, 1, 2, 4, 6 and 8 hr) and p‐IκBα, IκBα, p‐STAT3 and STAT3 expression were detected by Western blot. (b–c) Quantification of the Western blot analysis of p‐STAT3/STAT3 and p‐IκBα/IκBα. (d) RPE cells were preteated by 4 μM WP1066 for 1 hr and then 10 μg/L IL‐2 was added in serum‐free medium. (e–i) Quantification of the Western blot analysis (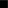, Control; 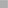, DMSO; 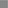, IL‐2 10 μg/L; 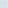, IL‐2 10 μg/L+WP1066; 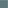, WP1066). (j) RPE cells were preteated by 10 μM BAY for 1 hr and then 10 μg/L IL‐2 was added in serum‐free medium. (k–o) Quantification of the Western blot analysis (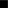, Control; 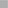, DMSO; 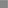, IL‐2 10 μg/L; 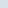, IL‐2 10 μg/L+BAY; 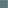, BAY). *p <* *.05, **p <* *.01

Previous studies have shown that IL‐2 can activate the JAK/STAT3 and NF‐κB signaling pathway in some cells (Fujii, 2007; Marzec et al., 2008). However, whether JAK/STAT3 and NF‐κB signaling pathway play important roles in RPE cells was not clear. Our results confirmed that IL‐2 could activate the JAK/STAT3 and NF‐κB signaling pathways in RPE cells (Figure 3a–c). Then we wondered whether the JAK/STAT3 and NF‐κB signaling pathways were involved in the IL‐2 induced expression of ECM proteins in RPE cells. RPE cells were treated with the JAK/STAT3 inhibitor WP1066 (4 μM) to block the JAK/STAT3 signaling pathway prior to IL‐2 for 2 hr. Consistent with prior observations, after treatment with 10 μg/L IL‐2, the protein expression levels of Fn, COL‐1 and TGF‐β2 were significantly increased compared with those in the control group. Nonetheless, the induction of Fn, COL‐1 and TGF‐β2 protein expression by IL‐2 was significantly inhibited by blocking the JAK/STAT3 signaling pathway with WP1066 or BAY (Figure 3d–i). Similarly, when BAY (10 μM) was used to block the NF‐κB signaling pathway, the induction of Fn, COL‐1 and TGF‐β2 protein expression by IL‐2 was significantly inhibited (Figure 3j–o). Intriguingly, either WP1066 or BAY were applied to block the specific pathway, the other pathway was blocked significantly (Figure 3i,n). These results might indicate that STAT3 and NK‐κB signaling pathways might interact with each other and played important roles in fibrosis in RPE cells together.

### 3.4 JAK/STAT3 and NF‐κB signaling pathways were involved in migration capability regulation

**Figure 4:**
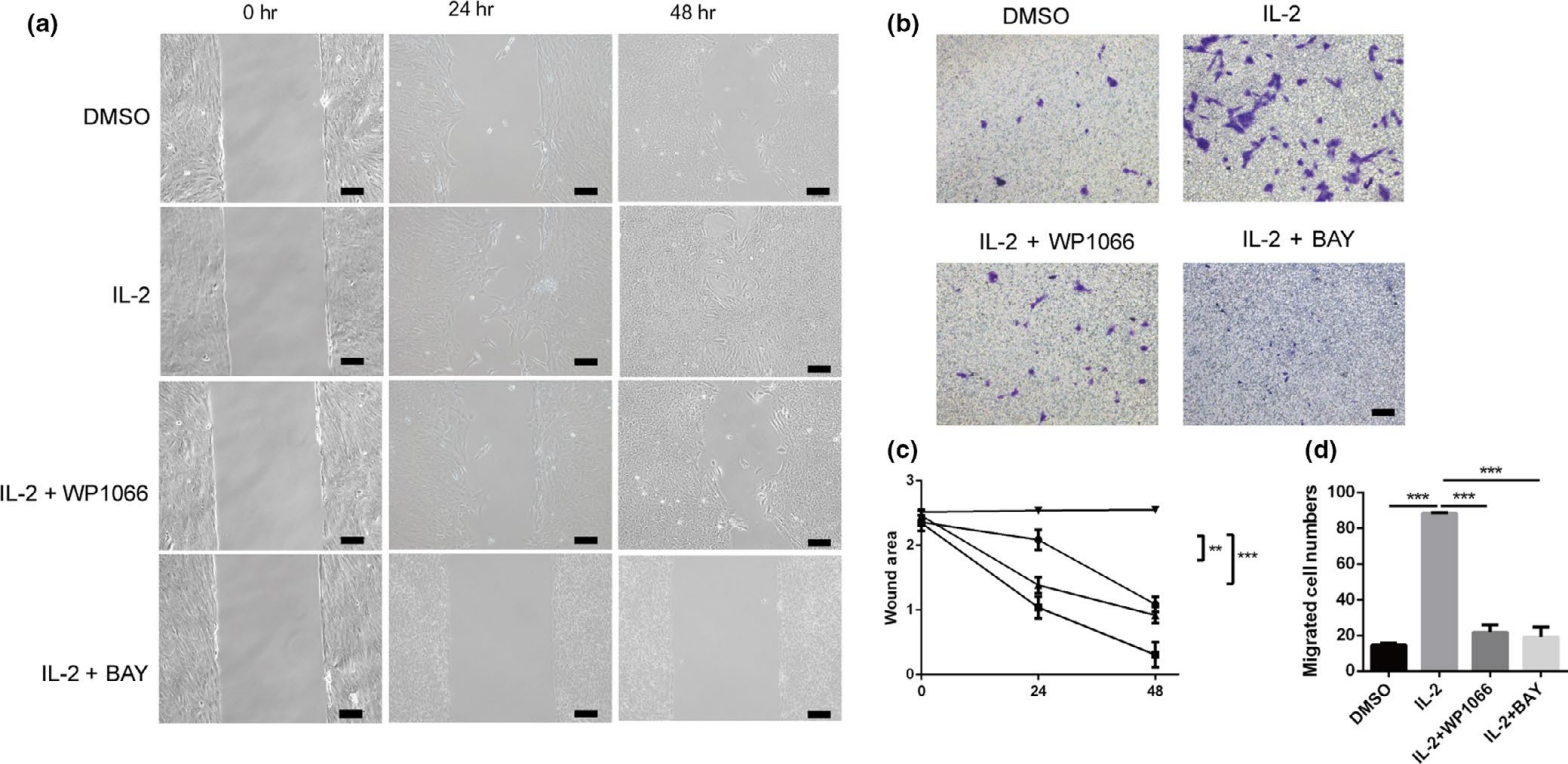
Both inhibition of JAK/STAT3 and NF‐κB signalling pathway lower migration rate. (a) RPE cells were pre‐seeded in the culture insert. When cells were 70% confluence, the insert was removed and serum‐free medium was added. Before 10 μg/L IL‐2 were loaded, WP1066 or BAY has been added into the medium for 1 hr. (b) Transwell assay of 10 μg/L IL‐2 and IL‐2 with 4 μM WP1066 or 10 μM BAY preteated for 1 hr. (c) The wound area was measured and analyzed with Image J software and there was a significance in 48 hr between the IL‐2 and IL‐2 with WP1066 or BAY group (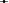, DMSO; 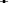, IL‐2; 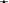, IL‐2+WP1066; 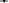, IL‐2+BAY). (d) Migrated RPE cells in DMSO group and IL‐2 with P1066 or BAY group respectively. **p <* *.01, ***p <* *.001. Scar bar: 100 μm

Cell migration is a central process in fibrosis disease (Anderluh, Kocic, Tomovic, Kocic, & Smelcerovic, 2019; Shelef, Bennin, Mosher, & Huttenlocher, 2012). Whether JAK/STAT3 and NF‐κB signaling pathways were involved in migration in RPE cells remain unknown. IL‐2 was identified to activate JAK/STAT3 and NF‐κB signaling pathways and furthermore increased ECM synthesis. Consistent with our results, IL‐2 could stimulate RPE cells migrate quickly. Our results indicated that with WP1066, the RPE cell migration rate is well below the IL‐2 group (*p *<* *.01, Figure 4a,c) during 48 hr. Similarly, the migration speed of IL‐2 with BAY was far lower (*p *<* *.001, Figure 4a,c) than that of IL‐2 group. In parallel, similar outcomes emerged in Transwell assay. The migrated cell in IL‐2 with WP1066 or BAY was far less than IL‐2 group (Figure 4b,d) after 24 hr. These results showed both JAK/STAT3 and NF‐κB pathways were involved in RPE cell migration induced by IL‐2.

## 4 DISCUSSION

Based on our experimental results, we hypothesized that the inflammatory microenvironment in the nAMD induced cytokines like IL‐2 expression. IL‐2 promoted RPE cells migration, ECM synthesis and TGF‐β2 expression by activating the STAT3 and NK‐κB signaling pathways but did not induce the expression of EMT‐specific proteins. Moreover, IL‐2 interacted with TGF‐β2 leading to ECM proteins expression excessively which aggravated macular fibrosis. In conclusion, IL‐2 played an important role on the fibrosis of macular degeneration. Anti‐inflammatory therapy might effectively prevent the fibrosis of macular in nAMD.

Fibrosis is a complicated pathophysiological process including cell migration, apoptosis, inflammation, EMT, ECM synthesis and so on (Chen et al., 2019; Li, Li, et al., 2019; Mack, 2018). As a pleiotropic inflammatory cytokine, IL‐2 is involved in cell proliferation, differentiation, immunoregulation and autoimmune or inflammatory diseases through the activation of intracellular signal transduction pathways (Klatzmann & Abbas, 2015). Several studies showed that IL‐2 could promote cell proliferation and migration (Llavero, Artaso, Lacerda, Parada, & Zugaza, 2016; Tauriainen et al., 2015). However, our results showed that IL‐2 could promote RPE cells migration but not proliferation (Figure S1A,B) in vitro. Considering cell proliferation and migration are two separate independent process and there was no proliferation pathways activated by IL‐2, IL‐2 may specifically promote RPE cells migration in nAMD rather than induce cell proliferation according our in vitro results. Whether IL‐2 could promote RPE cells proliferate directly or indirectly in vivo needs further research.

As a pleiotropic effects inflammatory factor, IL‐2 also plays an important role in promoting fibrosis (Taylor et al., 2018; Wang et al., 2016). IL‐2 plays important role in human nucleus pulposus cells migration and ECM synthesis (Wang et al., 2015). Moreover, a previous study showed that IL‐2 could mediate the EMT process through interacting with TGF‐β1 (Qian et al., 2017). However, whether IL‐2 could induce EMT and ECM synthesis in RPE cells and whether there is a relationship between this cytokine and TGF‐β2 remain unknown. According to the results, IL‐2 induced RPE cells ECM synthesis. Intriguingly, IL‐2 could not induce the expression of the EMT‐specific protein α‐SMA, which indicated that in RPE cells, IL‐2 might specifically promote the synthesis of ECM rather than induce EMT.

TGF‐β was considered a key member in the development of fibrosis. Our previous and several other studies found that IL‐1, IL‐6 and IL‐17A could enhance the effects of TGF‐β on fibrosis (Fabre, Kared, Friedman, & Shoukry, 2014; Ma, Yang, et al., 2018; Qian et al., 2017; Van Den Akker et al., 2017). A previous study showed anti‐VEGF could reduce TGF‐β1 expression but not cytokines in the aqueous humor of nAMD patients (Rezar‐Dreindl et al., 2016). Besides, our results showed IL‐2 could induce the expression of TGF‐β2 in RPE cells. These results indicated that cytokines could promote TGF‐β work. Inflammation might link to fibrosis in macular and nAMD relapse. However, the mechanism of interaction between IL‐2 and TGF‐β2 requires further study.

IL‐2 is involved in cell fibrosis, immunoregulation, development and other biological behaviors via activation of the STAT3 and NK‐κB signaling pathways (Jung et al., 2018; Luo et al., 2018; Mitrokhin, Gorbacheva, Mladenov, & Kamkin, 2018; Park et al., 2017; Wu et al., 2018). In our study, IL‐2 could activate STAT3 and NK‐κB signaling pathways. Moreover, WP1066 and BAY, the specific inhibitor of the STAT3 and NK‐κB signaling pathways, respectively, effectively suppressed the ECM synthesis induced by IL‐2 in RPE cells. These results indicate that the STAT3 and NK‐κB signaling pathways played important roles in IL‐2 induced ECM synthesis in RPE cells. Interestingly, Inhibition of STAT3 signaling pathway could suppress IκBα phosphorylation. Similarly, Inhibition of NK‐κB signaling pathway could suppress STAT3 phosphorylation. Because WP1066 could also partially inhibit ERK signaling according to the data sheet, herein we thought there might be key factors downstream of both JAK/STAT3 and NK‐κB function or other molecules regulated by signaling pathway inhibitors.

ECM is known to acquire substantial changes in composition, structure, and mechanics, which together exacerbate disease etiology through promotion of angiogenesis, cell differentiation, and immune activation (Shelef et al., 2012). Fn was verified to activate FAK consequently promote HLEC cell migration (Liu et al., 2018). Our results showed that with STAT3 and NF‐κB pathways inhibitors, the migration rate of RPE cells induced by IL‐2 dropped as well. So decreased ECM synthesis resulted from downregulated JAK/STAT3 and NF‐κB signaling pathways might reduce focal adhesion and thus migration capability.

These results might indicate that STAT3 and NK‐κB signaling pathways might interact with each other and played important roles in IL‐2‐induced fibrosis in RPE cells together. Our findings will offer new insights into our understanding of the molecular mechanisms underlying the pathogenesis of nAMD.

## CONFLICT OF INTEREST

All of the authors declare that there is no interest.

## References

[dgd12630-bib-0001] Anderluh, M. , Kocic, G. , Tomovic, K. , Kocic, H. , & Smelcerovic, A. (2019). DPP‐4 inhibition: Capital A, Cyrillic novel therapeutic approach to the treatment of pulmonary hypertension? Pharmacology & Therapeutics, 201, 1–7. 31095977 10.1016/j.pharmthera.2019.05.007

[dgd12630-bib-0002] Battaglia, A. , Buzzonetti, A. , Baranello, C. , Fanelli, M. , Fossati, M. , Catzola, V. , … Fattorossi, A. (2013). Interleukin‐21 (IL‐21) synergizes with IL‐2 to enhance T‐cell receptor‐induced human T‐cell proliferation and counteracts IL‐2/transforming growth factor‐beta‐induced regulatory T‐cell development. Immunology, 139, 109–120. 23278180 10.1111/imm.12061 PMC3634543

[dgd12630-bib-0003] Chambers, E. S. , Suwannasaen, D. , Mann, E. H. , Urry, Z. , Richards, D. F. , Lertmemongkolchai, G. , & Hawrylowicz, C. M. (2014). 1alpha,25‐dihydroxyvitamin D3 in combination with transforming growth factor‐beta increases the frequency of Foxp3(+) regulatory T cells through preferential expansion and usage of interleukin‐2. Immunology, 143, 52–60. 24673126 10.1111/imm.12289 PMC4137955

[dgd12630-bib-0004] Chen, Y. , Zhao, X. , Sun, J. , Su, W. , Zhang, L. , Li, Y. , … Liang, H. (2019). YAP1/Twist promotes fibroblast activation and lung fibrosis that conferred by miR‐15a loss in IPF. Cell Death and Differentiation, 26, 1832–1844. 30644438 10.1038/s41418-018-0250-0 PMC6748107

[dgd12630-bib-0005] Fabre, T. , Kared, H. , Friedman, S. L. , & Shoukry, N. H. (2014). IL‐17A enhances the expression of profibrotic genes through upregulation of the TGF‐beta receptor on hepatic stellate cells in a JNK‐dependent manner. Journal of Immunology, 193, 3925–3933. 10.4049/jimmunol.1400861 PMC4185218 25210118

[dgd12630-bib-0006] Fasler‐Kan, E. , Wunderlich, K. , Hildebrand, P. , Flammer, J. , & Meyer, P. (2005). Activated STAT 3 in choroidal neovascular membranes of patients with age‐related macular degeneration. Ophthalmologica, 219, 214–221. 16088240 10.1159/000085730

[dgd12630-bib-0007] Freudenberg, K. , Lindner, N. , Dohnke, S. , Garbe, A. I. , Schallenberg, S. , & Kretschmer, K. (2018). Critical Role of TGF‐beta and IL‐2 Receptor Signaling in Foxp3 Induction by an Inhibitor of DNA Methylation. Frontiers in Immunology, 9, 125. 29456534 10.3389/fimmu.2018.00125 PMC5801288

[dgd12630-bib-0008] Fujii, H. (2007). Cell type‐specific roles of Jak3 in IL‐2‐induced proliferative signal transduction. Biochemical and Biophysical Research Communications, 354, 825–829. 17266928 10.1016/j.bbrc.2007.01.067 PMC1839827

[dgd12630-bib-0009] Hendrayani, S. F. , Al‐Harbi, B. , Al‐Ansari, M. M. , Silva, G. , & Aboussekhra, A. (2016). The inflammatory/cancer‐related IL‐6/STAT3/NF‐kappaB positive feedback loop includes AUF1 and maintains the active state of breast myofibroblasts. Oncotarget, 7, 41974–41985. 27248826 10.18632/oncotarget.9633 PMC5173109

[dgd12630-bib-0010] Hseu, Y. C. , Lin, Y. C. , Rajendran, P. , Thigarajan, V. , Mathew, D. C. , Lin, K. Y. , … Yang, H. L. (2019). Antrodia salmonea suppresses invasion and metastasis in triple‐negative breast cancer cells by reversing EMT through the NF‐kappaB and Wnt/beta‐catenin signaling pathway. Food and Chemical Toxicology, 124, 219–230. 30529123 10.1016/j.fct.2018.12.009

[dgd12630-bib-0011] Jung, K. B. , Lee, H. , Son, Y. S. , Lee, M. O. , Kim, Y.‐D. , Oh, S. J. , … Son, M.‐Y. (2018). Interleukin‐2 induces the in vitro maturation of human pluripotent stem cell‐derived intestinal organoids. Nature Communications, 9, 3039. 10.1038/s41467-018-05450-8 PMC6072745 30072687

[dgd12630-bib-0012] Jung, K. I. , Woo, J. E. , & Park, C. K. (2019). Effects of aqueous suppressants and prostaglandin analogues on early wound healing after glaucoma implant surgery. Scientific Reports, 9, 5251. 30918313 10.1038/s41598-019-41790-1 PMC6437192

[dgd12630-bib-0013] Kauppinen, A. , Paterno, J. J. , Blasiak, J. , Salminen, A. , & Kaarniranta, K. (2016). Inflammation and its role in age‐related macular degeneration. Cellular and Molecular Life Sciences, 73, 1765–1786. 26852158 10.1007/s00018-016-2147-8 PMC4819943

[dgd12630-bib-0014] Kim, M. S. , Lee, H. S. , Kim, Y. J. , Lee, D. Y. , Kang, S. G. , & Jin, W. (2019). MEST induces Twist‐1‐mediated EMT through STAT3 activation in breast cancers. Cell Death and Differentiation, 26. 10.1038/s41418-019-0322-9 PMC7224286 30903102

[dgd12630-bib-0015] Klatzmann, D. , & Abbas, A. K. (2015). The promise of low‐dose interleukin‐2 therapy for autoimmune and inflammatory diseases. Nature Reviews Immunology, 15, 283–294. 10.1038/nri3823 25882245

[dgd12630-bib-0016] Kutty, R. K. , Samuel, W. , Duncan, T. , Postnikova, O. , Jaworski, C. , Nagineni, C. N. , & Redmond, T. M. (2018). Proinflammatory cytokine interferon‐gamma increases the expression of BANCR, a long non‐coding RNA, in retinal pigment epithelial cells. Cytokine, 104, 147–150. 29054724 10.1016/j.cyto.2017.10.009 PMC5847440

[dgd12630-bib-0017] Li, L. , Li, Q. , Wei, L. , Wang, Z. , Ma, W. , Liu, F. , … Qian, Y. (2019). Chemokine (C‐X‐C motif) ligand 14 contributes to lipopolysaccharide‐induced fibrogenesis in mouse L929 fibroblasts via modulating PPM1A. Journal of Cellular Biochemistry, 120, 13372–13381. 30920024 10.1002/jcb.28612

[dgd12630-bib-0018] Li, L. , Xu, J. , Qiu, G. , Ying, J. , Du, Z. , Xiang, T. , … Tao, Q. (2018). Epigenomic characterization of a p53‐regulated 3p22.2 tumor suppressor that inhibits STAT3 phosphorylation via protein docking and is frequently methylated in esophageal and other carcinomas. Theranostics, 8, 61–77. 29290793 10.7150/thno.20893 PMC5743460

[dgd12630-bib-0019] Li, Q. , Ye, W. X. , Huang, Z. J. , Zhang, Q. , & He, Y. F. (2019). Effect of IL‐6‐mediated STAT3 signaling pathway on myocardial apoptosis in mice with dilated cardiomyopathy. European Review for Medical and Pharmacological Sciences, 23, 3042–3050. 31002169 10.26355/eurrev_201904_17586

[dgd12630-bib-0020] Lin, T. , Walker, G. B. , Kurji, K. , Fang, E. , Law, G. , Prasad, S. S. , … Matsubara, J. A. (2013). Parainflammation associated with advanced glycation endproduct stimulation of RPE in vitro: Implications for age‐related degenerative diseases of the eye. Cytokine, 62, 369–381. 23601964 10.1016/j.cyto.2013.03.027 PMC3947380

[dgd12630-bib-0021] Little, K. , Ma, J. H. , Yang, N. , Chen, M. , & Xu, H. (2018). Myofibroblasts in macular fibrosis secondary to neovascular age‐related macular degeneration ‐ the potential sources and molecular cues for their recruitment and activation. EBioMedicine, 38, 283–291. 30473378 10.1016/j.ebiom.2018.11.029 PMC6306402

[dgd12630-bib-0022] Liu, J. , Xu, D. , Li, J. , Gao, N. , Liao, C. , Jing, R. , … Pei, C. (2018). The role of focal adhesion kinase in transforming growth factor‐beta2 induced migration of human lens epithelial cells. International Journal of Molecular Medicine, 42, 3591–3601. 30280182 10.3892/ijmm.2018.3912

[dgd12630-bib-0023] Llavero, F. , Artaso, A. , Lacerda, H. M. , Parada, L. A. , & Zugaza, J. L. (2016). Lck/PLCgamma control migration and proliferation of interleukin (IL)‐2‐stimulated T cells via the Rac1 GTPase/glycogen phosphorylase pathway. Cellular Signalling, 28, 1713–1724. 27519475 10.1016/j.cellsig.2016.07.014

[dgd12630-bib-0024] Luo, J. , Ming, B. , Zhang, C. , Deng, X. , Li, P. , Wei, Z. , … Dong, L. (2018). IL‐2 inhibition of Th17 generation rather than induction of Treg cells is impaired in primary Sjogren's syndrome patients. Frontiers in Immunology, 9, 1755. 30150979 10.3389/fimmu.2018.01755 PMC6100298

[dgd12630-bib-0025] Ma, B. , Jing, R. , Liu, J. , Yang, L. , Li, J. , Qin, L. , … Pei, C. (2018). CTGF contributes to the development of posterior capsule opacification: An in vitro and in vivo study. International Journal of Biological Sciences, 14, 437–448. 29725265 10.7150/ijbs.23946 PMC5930476

[dgd12630-bib-0026] Ma, B. , Yang, L. , Jing, R. , Liu, J. , Quan, Y. , Hui, Q. , … Pei, C. (2018). Effects of Interleukin‐6 on posterior capsular opacification. Experimental Eye Research, 172, 94–103. 29617629 10.1016/j.exer.2018.03.013

[dgd12630-bib-0027] Mack, M. (2018). Inflammation and fibrosis. Matrix Biology, 68–69, 106–121. 10.1016/j.matbio.2017.11.010 29196207

[dgd12630-bib-0028] Makarev, E. , Cantor, C. , Zhavoronkov, A. , Buzdin, A. , Aliper, A. , & Csoka, A. B. (2014). Pathway activation profiling reveals new insights into age‐related macular degeneration and provides avenues for therapeutic interventions. Aging, 6, 1064–1075. 25543336 10.18632/aging.100711 PMC4298366

[dgd12630-bib-0029] Marzec, M. , Halasa, K. , Kasprzycka, M. , Wysocka, M. , Liu, X. , Tobias, J. W. , … Wasik, M. A. (2008). Differential effects of interleukin‐2 and interleukin‐15 versus interleukin‐21 on CD4 + cutaneous T‐cell lymphoma cells. Cancer Research, 68, 1083–1091. 18281483 10.1158/0008-5472.CAN-07-2403

[dgd12630-bib-0030] Mitchell, P. , Liew, G. , Gopinath, B. , & Wong, T. Y. (2018). Age‐related macular degeneration. Lancet, 392, 1147–1159. 30303083 10.1016/S0140-6736(18)31550-2

[dgd12630-bib-0031] Mitrokhin, V. , Gorbacheva, L. , Mladenov, M. , & Kamkin, A. (2018). IL‐2‐induced NF‐kappaB phosphorylation upregulates cation nonselective conductance in human cardiac fibroblasts. International Immunopharmacology, 64, 170–174. 30189394 10.1016/j.intimp.2018.08.040

[dgd12630-bib-0032] Newman, A. M. , Gallo, N. B. , Hancox, L. S. , Miller, N. J. , Radeke, C. M. , Maloney, M. A. , … Radeke, M. J. (2012). Systems‐level analysis of age‐related macular degeneration reveals global biomarkers and phenotype‐specific functional networks. Genome Medicine, 4, 16. 22364233 10.1186/gm315 PMC3372225

[dgd12630-bib-0033] Park, J. U. , Kang, B. Y. , Lee, H. J. , Kim, S. , Bae, D. , Park, J. H. , & Kim, Y. R. (2017). Tetradecanol reduces EL‐4 T cell growth by the down regulation of NF‐kappaB mediated IL‐2 secretion. European Journal of Pharmacology, 799, 135–142. 28167257 10.1016/j.ejphar.2017.02.002

[dgd12630-bib-0034] Qian, Y. , Yao, W. , Yang, T. , Yang, Y. , Liu, Y. , Shen, Q. , … Wang, J. (2017). aPKC‐iota/P‐Sp1/Snail signaling induces epithelial‐mesenchymal transition and immunosuppression in cholangiocarcinoma. Hepatology, 66, 1165–1182. 28574228 10.1002/hep.29296

[dgd12630-bib-0035] Rezar‐Dreindl, S. , Sacu, S. , Eibenberger, K. , Pollreisz, A. , Bühl, W. , Georgopoulos, M. , … Schmidt‐Erfurth, U. (2016). The intraocular cytokine profile and therapeutic response in persistent neovascular age‐related macular degeneration. Investigative Ophthalmology & Visual Science, 57, 4144–4150. 27537264 10.1167/iovs.16-19772

[dgd12630-bib-0036] Ricker, L. J. , Kijlstra, A. , Kessels, A. G. , de Jager, W. , Liem, A. T. A. , Hendrikse, F. , & La Heij, E. C. (2011). Interleukin and growth factor levels in subretinal fluid in rhegmatogenous retinal detachment: A case‐control study. PLoS ONE, 6, e19141. 21556354 10.1371/journal.pone.0019141 PMC3083411

[dgd12630-bib-0037] Roh, M. I. , Kim, H. S. , Song, J. H. , Lim, J. B. , Koh, H. J. , & Kwon, O. W. (2009). Concentration of cytokines in the aqueous humor of patients with naive, recurrent and regressed CNV associated with amd after bevacizumab treatment. Retina, 29, 523–529. 19262441 10.1097/IAE.0b013e318195cb15

[dgd12630-bib-0038] Roybal, C. N. , Velez, G. , Toral, M. A. , Tsang, S. H. , Bassuk, A. G. , & Mahajan, V. B. (2018). Personalized proteomics in proliferative vitreoretinopathy implicate hematopoietic cell recruitment and mTOR as a therapeutic target. American Journal of Ophthalmology, 186, 152–163. 29246578 10.1016/j.ajo.2017.11.025 PMC5805631

[dgd12630-bib-0039] Sakurada, Y. , Nakamura, Y. , Yoneyama, S. , Mabuchi, F. , Gotoh, T. , Tateno, Y. , … Iijima, H. (2015). Aqueous humor cytokine levels in patients with polypoidal choroidal vasculopathy and neovascular age‐related macular degeneration. Ophthalmic Research, 53, 2–7. 25472810 10.1159/000365487

[dgd12630-bib-0040] Shelef, M. A. , Bennin, D. A. , Mosher, D. F. , & Huttenlocher, A. (2012). Citrullination of fibronectin modulates synovial fibroblast behavior. Arthritis Research & Therapy, 14, R240. 23127210 10.1186/ar4083 PMC3674601

[dgd12630-bib-0041] Shen, Y. , Xie, C. , Gu, Y. , Li, X. , & Tong, J. (2016). Illumination from light‐emitting diodes (LEDs) disrupts pathological cytokines expression and activates relevant signal pathways in primary human retinal pigment epithelial cells. Experimental Eye Research, 145, 456–467. 26432918 10.1016/j.exer.2015.09.016

[dgd12630-bib-0042] Solomon, S. D. , Lindsley, K. , Vedula, S. S. , Krzystolik, M. G. , & Hawkins, B. S. (2019). Anti‐vascular endothelial growth factor for neovascular age‐related macular degeneration. The Cochrane Database of Systematic Reviews, 3, Cd005139. 30834517 10.1002/14651858.CD005139.pub4 PMC6419319

[dgd12630-bib-0043] Tauriainen, J. , Gustafsson, K. , Gothlin, M. , Gertow, J. , Buggert, M. , Frisk, T. W. , … Önfelt, B. (2015). Single‐cell characterization of in vitro migration and interaction dynamics of T cells expanded with IL‐2 and IL‐7. Frontiers in Immunology, 6, 196. 25972868 10.3389/fimmu.2015.00196 PMC4412128

[dgd12630-bib-0044] Taylor, A. E. , Carey, A. N. , Kudira, R. , Lages, C. S. , Shi, T. , Lam, S. , … Miethke, A. G. (2018). Interleukin 2 promotes hepatic regulatory T cell responses and protects from biliary fibrosis in murine sclerosing cholangitis. Hepatology, 68, 1905–1921. 29698570 10.1002/hep.30061 PMC6203671

[dgd12630-bib-0045] Tischner, D. , Wiegers, G. J. , Fiegl, H. , Drach, M. , & Villunger, A. (2012). Mutual antagonism of TGF‐beta and Interleukin‐2 in cell survival and lineage commitment of induced regulatory T cells. Cell Death and Differentiation, 19, 1277–1287. 22322859 10.1038/cdd.2012.7 PMC3392638

[dgd12630-bib-0046] Van Den Akker, G. G. , Van Beuningen, H. M. , Vitters, E. L. , Koenders, M. I. , van de Loo, F. A. , van Lent, P. L. , … van der Kraan, P. M. (2017). Interleukin 1 beta‐induced SMAD2/3 linker modifications are TAK1 dependent and delay TGFbeta signaling in primary human mesenchymal stem cells. Cellular Signalling, 40, 190–199. 28943409 10.1016/j.cellsig.2017.09.010

[dgd12630-bib-0047] Wang, H. , Hou, L. , Kwak, D. , Fassett, J. , Xu, X. , Chen, A. , & Chen, Y. (2016). Increasing regulatory T cells with Interleukin‐2 and Interleukin‐2 antibody complexes attenuates lung inflammation and heart failure progression. Hypertension, 68, 114–122. 27160197 10.1161/HYPERTENSIONAHA.116.07084 PMC5022287

[dgd12630-bib-0048] Wang, K. , Li, H. , Sun, R. , Liu, C. , Luo, Y. , Fu, S. , & Ying, Y. (2019). Emerging roles of transforming growth factor beta signaling in wet age‐related macular degeneration. Acta Biochimica et Biophysica Sinica, 51, 1–8. 30496406 10.1093/abbs/gmy145

[dgd12630-bib-0049] Wang, Z. , Wang, G. , Zhu, X. , Geng, D. , & Yang, H. (2015). Interleukin‐2 is upregulated in patients with a prolapsed lumbar intervertebral disc and modulates cell proliferation, apoptosis and extracellular matrix metabolism of human nucleus pulposus cells. Experimental and Therapeutic Medicine, 10, 2437–2443. 26668654 10.3892/etm.2015.2809 PMC4665125

[dgd12630-bib-0050] Wu, D. M. , Wen, X. , Wang, Y. J. , Han, X. R. , Wang, S. , Shen, M. , & Zheng, Y. L. (2018). Effect of microRNA‐186 on oxidative stress injury of neuron by targeting interleukin 2 through the janus kinase‐signal transducer and activator of transcription pathway in a rat model of Alzheimer's disease. Journal of Cellular Physiology, 233, 9488–9502. 29995978 10.1002/jcp.26843

[dgd12630-bib-0051] Yamamoto, H. , Fara, A. F. , Dasgupta, P. , & Kemper, C. (2013). CD46: The ‘multitasker’ of complement proteins. International Journal of Biochemistry & Cell Biology, 45, 2808–2820. 24120647 10.1016/j.biocel.2013.09.016

[dgd12630-bib-0052] Yonekawa, Y. , & Kim, I. K. (2014). Clinical characteristics and current treatment of age‐related macular degeneration. Cold Spring Harbor Perspectives in Medicine, 5, a017178. 25280900 10.1101/cshperspect.a017178 PMC4292078

[dgd12630-bib-0053] Zhang, Y. , & Huang, W. (2018). Transforming growth factor beta1 (TGF‐beta1)‐Stimulated Integrin‐Linked Kinase (ILK) regulates migration and epithelial‐mesenchymal transition (EMT) of human lens epithelial cells via nuclear factor kappaB (NF‐kappaB). Medical Science Monitor, 24, 7424–7430. 30332398 10.12659/MSM.910601 PMC6201705

