# Supplementary material for: Interleukin‐2 induces extracellular matrix synthesis and TGF‐β2 expression in retinal pigment epithelial cells
Source: Dev Growth Differ. 2019 Oct 13;61(7-8):410–8. doi: 10.1111/dgd.12630 (PMC6899885; doi:10.1111/dgd.12630)
Supplement: Supplementary file 1 [file DGD-61-410-s001.docx]

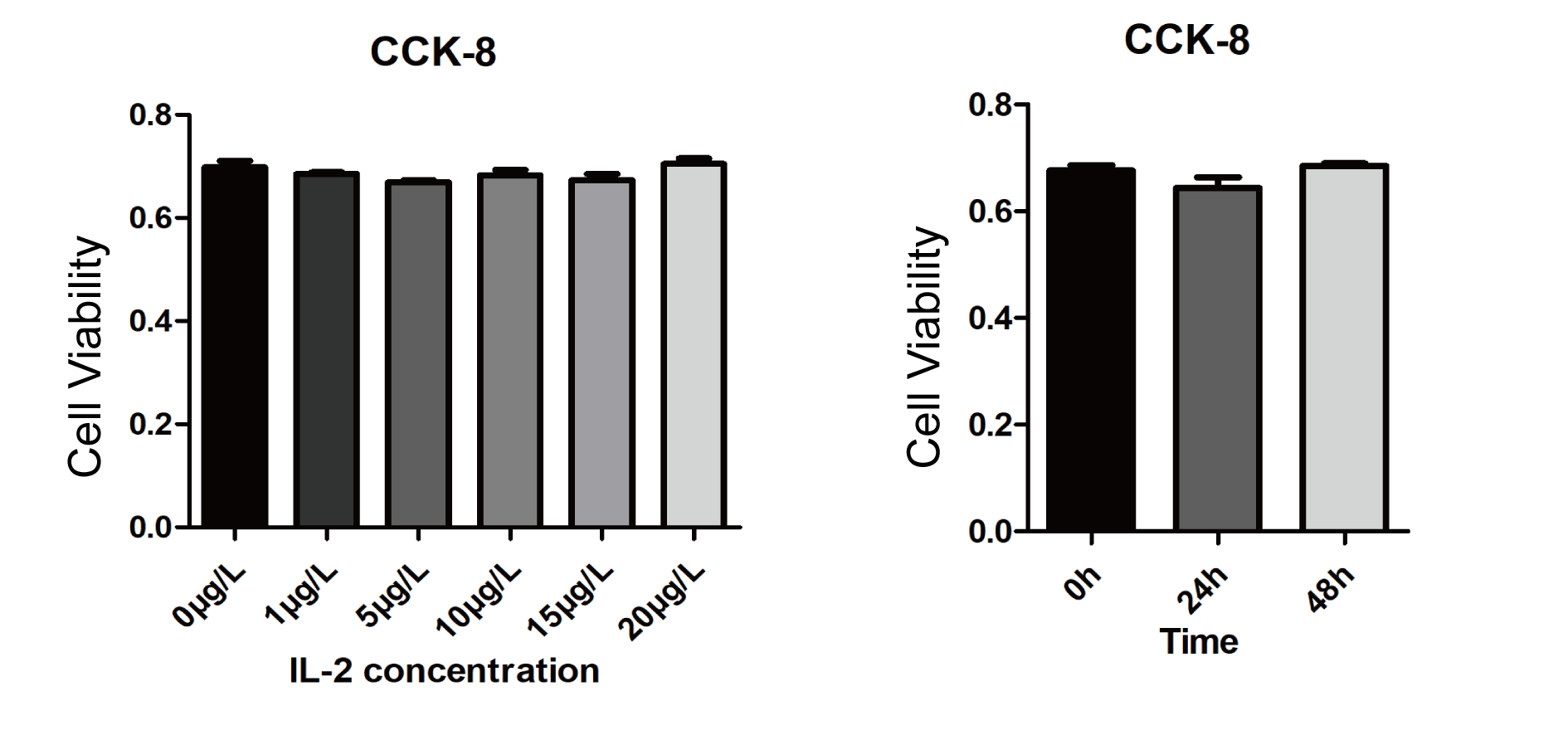


**Fig S1 toxicity experiments of IL-2 on RPE cells.** A: RPE cells were treated by 0, 1, 5, 10, 15, 20 μg/L IL-2 for 24 h and CCK8 probe was used to measure the OD at 450 nm. B: RPE cells were treated by 10 μg/L IL-2 for 0, 24, 48 h and CCK8 probe was used to measure the OD at 450 nm. There was no difference between groups.
